# Guanidine acetic acid exhibited greater growth performance in younger (13–30 kg) than in older (30–50 kg) lambs under high-concentrate feedlotting pattern

**DOI:** 10.3389/fvets.2022.954675

**Published:** 2022-08-04

**Authors:** Wen-Juan Li, Qi-Chao Wu, Zhao-Yang Cui, Yao-Wen Jiang, Ailiyasi Aisikaer, Fan Zhang, He-Wei Chen, Wei-Kang Wang, Yan-Lu Wang, Liang-Kang Lv, Feng-Liang Xiong, Ying-Yi Liu, Sheng-Li Li, Hong-Jian Yang

**Affiliations:** State Key Laboratory of Animal Nutrition, College of Animal Science and Technology, China Agricultural University, Beijing, China

**Keywords:** UGAA, CGAA, forage type, nutrient digestion, antioxidant index

## Abstract

Guanidine acetic acid (GAA) is increasingly considered as a nutritional growth promoter in monogastric animals. Whether or not such response would exist in rapid-growing lambs is unclear yet. The objective of this study was to investigate whether dietary supplementation with uncoated GAA (UGAA) and coated GAA (CGAA) could alter growth performance, nutrient digestion, serum metabolites, and antioxidant capacity in lambs. Seventy-two small-tailed *Han* lambs initially weighed 12 ± 1.6 kg were randomly allocated into six groups in a 2 × 3 factorial experimental design including two forage-type rations [Oaten hay (OH) *vs*. its combination with wheat silage (OHWS)] and three GAA treatment per ration: no GAA, 1 g UGAA, and 1 g CGAA per kg dry matter. The whole experiment was completed in two consecutive growing stages (stage 1, 13–30 kg; stage 2, 30–50 kg). Under high-concentrate feeding pattern (Stage 1, 25: 75; Stage 2, 20: 80), UGAA or CGAA supplementation in young lambs presented greater dry matter intake (DMI) in stage 1 and average daily gain (ADG) in the whole experimental period; lambs in OH group had higher ADG and DMI than that in OHWS group in stage 1 and whole experimental period, but this phenomenon was not observed in stage 2. Both UCGA and CGAA addition increased dietary DM, organic matter (OM), neutral detergent fiber (NDF), and acid detergent fiber (ADF) digestion in both stages. In blood metabolism, UCGA and CGAA addition resulted in a greater total protein (TP) and insulin-like growth factor 1(IGF-1) levels, as well as antioxidant capacity; at the same time, UCGA and CGAA addition increased GAA metabolism-creatine kinase and decreased guanidinoacetate N-methyltransferase (GAMT) and *L*-Arginine glycine amidine transferase catalyzes (AGAT) activity. In a brief, the results obtained in the present study suggested that GAA (UGAA and CGAA; 1 g/kg DM) could be applied to improve growth performance in younger (13–30 kg) instead of older (30–50 kg) lambs in high-concentrate feedlotting practice.

## Introduction

**Figure 1:**
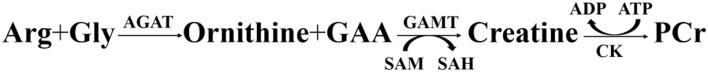
Metabolic processes of GAA. (AGAT, *L*-arginine: glycine amidinotransferase; Arg, arginine; CK, creatine kinase; GAMT, guanidinoacetate N-methyltransferase; Gly, glycine; SAH, S-Adenosine homocysteine; SAM, S-Adenosylmethionine; PCr, phosphocreatine).

Guanidinoacetic acid (GAA) is a natural primary precursor of creatine and synthesized in the pancreas and kidney from arginine and glycine under the catalysis of *L*-arginine: glycine amidinotransferase (AGAT). Afterward, S-adenosylmethionine (SAM) methylated GAA to form creatine, and the latter participated in energy and protein metabolism (1–3), completing GAA biotransformation (see Figure 1). In an earlier *in vitro* study (4), about 1.7% of the creatine and phosphocreatine pool was irreversibly converted to creatinine each day and excreted in the urine. Borosnan et al. (5) noted that creatine was essential for animal body to maintain permanent muscle growth, and animal body itself was able to compensate for the creatine loss *via* the decomposition of creatine to creatinine, and the latter excreted in urine. Therefore, the author in that study (6) speculated that the requirement for creatine was likely greater in young growing animals than in adult animals, and as aforementioned compensation for creatine losses, it might be necessary to provide extra creatine to the growing tissues.

Previous studies reported that dietary GAA addition (0, 0.6, 6 g/kg basal diet, day-old male broiler; 0, 0.6 g/kg basal diet, 25-day-old broiler; 0, 0.8, 1.2 g/kg basal diet, weaned pig; 0, 0.3, 0.6, 0.9 g/kg basal diet, finishing pig) presented beneficial effects on monogastric animals in terms of average daily gain (ADG), slaughter characteristics and carcass meat quality (7–10). In ruminant animals, limited studies indicated that dietary GAA addition 0 or 0.6 g/kg basal diet improved growth performance, as well as ruminal fermentation, and apparent total tract nutrient digestibility in yearling Angus bulls with a mean body weight of 440 kg (11, 12). Unlike monogastric animals, the application of GAA in ruminants should not neglect its microbial degradation in the rumen. Speer et al. (13) in a recent study noted that the ruminal degradation rate of GAA was 0.47–0.49 before it was absorbed in the lower digestive tract. For ruminants, the type of forage (e.g., hay and silage) is one of the factors that affect growth performance (14, 15). Therefore, the objective of the present study was to elucidate whether dietary supplementation of CGAA in comparison with UGAA could exhibit greater growth performance in feedlotting lambs with two forage types, and what possible action mode could exist for GAA during the decomposition of creatine to creatinine.

## Materials and methods

The feeding trial in the present study was conducted at a sheep farm at Huanghua (38°22'N, 117°31'E, Cangzhou, Hebei province, China) from December 1, 2020 to March 31, 2021. The animals were kept in an enclosed animal house and the mean minimum and maximum room temperatures observed during the experimental period were −20°C and 23°C (average 0.9°C), respectively. In the present study, all the procedures performed in animal feeding and sample collection followed the Guidelines of the Beijing Municipal Council on Animal Care (with protocolCAU20171014-1).

### Guanidinoacetic acid products

The uncoated guanidino acetic acid (UGAA) and coated guanidino acetic acid (CGAA) used in this trail had the form of a white powder with available content of 984 g/kg and 600 g/kg GAA, respectively. Among them, the packaging material coating with CGAA is mainly fat powder. UGAA and CGAA were provided by Hebei Guang rui Company, Shijiazhuang city, Hebei province, China.

### Experimental design and animal feeding management

Seventy-two small-tailed male Chinese *Han* lambs initially weighed 12 ± 1.6 kg of BW were chosen as experimental animals and fed total mixed rations with different forage: concentrate ratios (Stage 1, 25: 75; Stage 2, 20: 80) on dry matter basis except that the forage source was different as listed in Table 1.

**Table 1:** Nutrient composition of oaten hay and wheat silage on dry matter basis.

**Nutrient level**	**DM(g/kg)**	**CP(g/kg)**	**EE(g/kg)**	**NDF (g/kg)**	**ADF (g/kg)**	**OM(g/kg)**	**GE(MJ/kg)**
Oaten hay (OH)	879.5	118.0	24.1	611.6	351.4	917.0	14.4
Wheat silage (WS)	329.3	120.8	32.0	520.0	310.5	919.0	14.2

ADF, acid detergent fiber; CP, crude protein; DM, dry matter; EE, ether extract; GE, gross energy; NDF, neutral detergent fiber; OM, organic matter.

A 2 × 3 factorial experiment design was applied to randomly allocate the animals into two forage type of TMRs (OH, rations with sole forage of oaten hay; OHWS, rations with forage combination of wheat silage and oaten hay) and three GAA addition group within each above forage type of ration (control, a basal control ration without GAA addition; UGAA, a basal ration added with 1.0 g/kg uncoated GAA; CGAA, a basal ration added with 1.0 g/kg coated GAA). Consequently, the aforementioned experimental design resulted in six ration treatments, and each treatment had four bamboo-slotted bedding pens and each pen was arranged with three lambs with free access to drinking water. Prior to ration feeding, the corresponding amounts of UGAA or CGAA were mixed with concentrate feeds first and then fed with corresponding type of forage following the experimental design. The whole experimental trial lasted for 127 days consisting of 7 days adaptation and 120 days for data collection with each stage about 60 days. All the rations were formulated to satisfy nutrient requirement of 300 g gain/day (16) and all lambs were fed twice at 0800 and 1,600 h daily (Table 2).

**Table 2:** Ingredient and chemical composition of total mixed rations with different forage type fed to lambs at different feeding stages (stage 1, 1–62 day; stage 2, 63–120 day).

**Ingredients**	**Stage 1**		**Stage 2**	
	**OH**	**OHWS**	**OH**	**OHWS**
Wheat silage	0	90	0	70
Oaten hay	250	160	200	130
Concentrate^a^, ^b^	750	750	800	800
Nutrient level (g/kg, as Dry Matter)^c^				
Organic matter	893.3	901.8	905.6	910.5
Crude protein	191.9	193.7	184.6	174.5
Ether extract	36.6	35.1	38	37.5
Neutral detergent fiber	307.6	293.8	274.5	271.2
Acid detergent fiber	118.4	117.1	108	99.7
Gross energy (MJ/kg)	15.4	15.5	17	16.8

OH, the rations with the forage type of oaten hay; OHWS, the rations with the forage type of oaten hay plus wheat silage.

a Contained per kg in stage 1: 380 g Corn meal, 150 g Soybean meal, 180 g DDGS, 40 g Premix, Contained per kg in stage 2: 500 g Corn meal, 150 g Soybean meal, 110 g DDGS, 40 g Premix.

b Contained per kg premix:200–500 mg Cu, 750–17500 mg Fe, 750–5,000 mg Mn, 1,250–4,250 mg Zn, 3.75–350 mg I, 2.5–17.87 mg Se, 75000–350000 IU vitamin A, 12,500–142,500 IU vitamin D_3_, 500 mg vitamin E.

c Determined using samples pooled by diet three times within each week.

### Growth performance

During the feeding trial period, live body weight of each animal was weighed before morning feeding and recorded on days 1, 18, 33, 47, 62, 90, and 120 to calculate ADG at above different feeding intervals. The ration amounts offered were daily recorded, and the ration leftovers were weighed before next day's morning feeding to calculate actual feed intake per pen level. The moisture content of total mixed ration was weekly determined and adjusted to calculate dry matter intake (DMI). Feed conversion efficiency was expressed as F: G ratio and calculated as DMI divided by ADG.

### Apparent nutrient digestibility

At the end of each feeding stage, fecal samples per animal were collected *via* the rectum for three consecutive days after morning feeding. Meanwhile, representative samples of ration offered, orts, and feces were oven-dried at 65°C for 48 h and ground to pass a 1 mm sieve. Acid-insoluble ash (AIA) was measured following the method of Van-Keulen and Young (17). Dry matter (DM), organic matter (OM), ether extract (EE), crude protein (CP), and acid detergent fiber (ADF) were analyzed following the analysis procedures of AOAC (18). Neutral detergent fiber (NDF) was assayed following the method of Van Soest et al. (19). Apparently, total tract digestibility of nutrients was calculated as follow:


$$\begin{array}{l} Nutrient\;digestibility\;(\%)\\ \;=100-100\times (N_{f}\times R_{AIA})/(N_{r}\times F_{AIA}) \end{array}$$

where *N*_*f*_ is nutrient concentration in feces, *R*_*AIA*_ is AIA content in rations, *N*_*r*_ is nutrient content in rations, and *F*_*AIA*_ is AIA content in feces.

### Blood sample analysis

On days 60 and 120, blood samples of each animal were collected *via* the coccygeal vein into the 5-mL evacuated tubes before the morning meal. The blood samples kept overnight at 4°C were centrifuged at 3,000 g for 10 min to obtain serum samples. Afterward, the serum samples were sent to a third-party examination agency (Shanghai Kehua Biology Science and Technology Co., Ltd; Shanghai, China) and subjected to the determination of total protein (TP), albumin (ALB), urea N, triglycerides (TG), glucose, insulin-like growth factor 1 (IGF-1), creatine kinase (CK), creatinine, GAMT, AGAT, adenosine Triphosphate (ATP), superoxide dismutase (SOD), malondialdehyde (MDA), catalase (CAT), glutathione peroxidase (GSH-*PX*), total antioxidant Capacity (T-AOC), and glutathione (GSH) using the corresponding enzyme-linked immunosorbent assay kit. The contents of GAA and creatine in blood were determined with high-performance liquid chromatography (HPLC) as described by Buchberger and Ferdig (20).

### Statistical analysis

Data of each lamb feeding stage (stage 1 and stage 2) were analyzed using the MIXED procedure of the Statistical Analysis System Institute. The model was applied as follows:


$$\begin{array}{l} Y_{ijk}=\mu +G_{i}+F_{j}+{\left(G\times F\right)}_{ij}+R_{k}+e_{ijk} \end{array}$$

where *Y*_*ijk*_ is the dependent variable, μ is the overall mean, *G*_*i*_ is the fixed effect of GAA addition (*I* = 3: Control, UGAA, CGAA), *F*_*j*_ is the fixed effect of total mixed ration type with different forage source (OH, oaten hay; OHWS, oaten hay plus wheat silage), G × F is the interaction of GAA and ration type. *R*_*k*_ is the random effect of animals (*k* = 12 per treatment) or pens (*k* = 4 per treatment), and *e*_*ijk*_ is residue error term. Least square means and standard errors of the means were calculated with the LSMEANS statement of the SAS software. Significance was declared at *P* ≤ 0.05 unless otherwise noted.

## Results

### Effect of forage type and GAA addition on growth performance and feed efficiency

**Figure 2:**
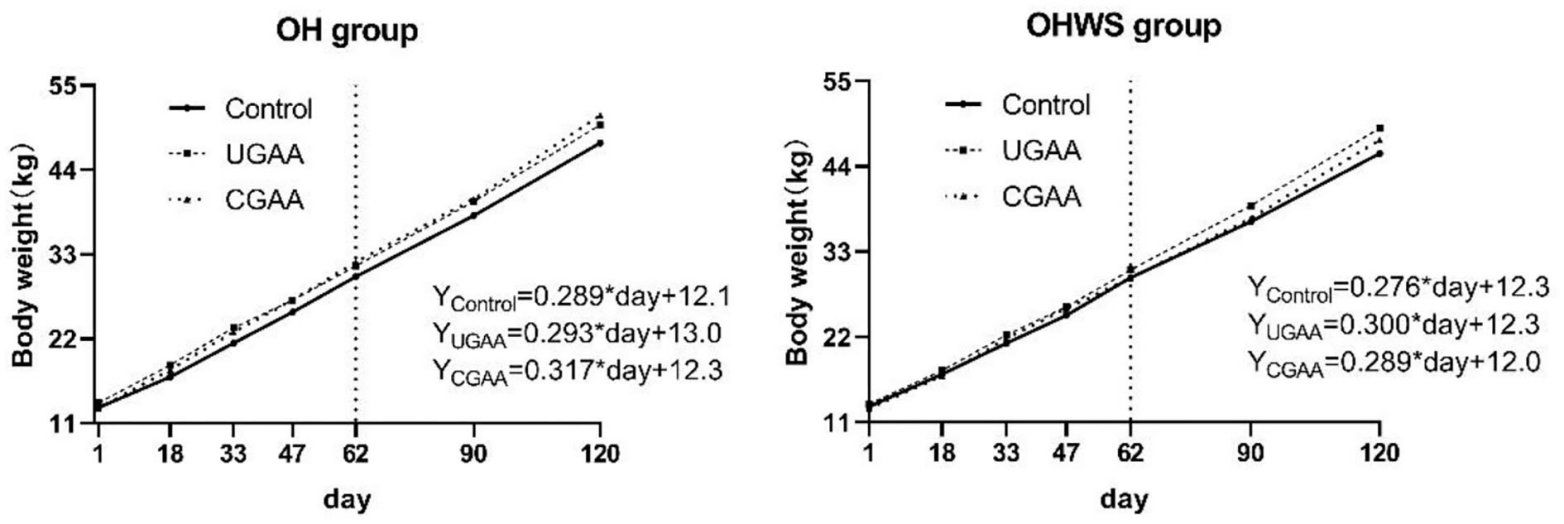
The effect of forage type and guanidine acetic acid (GAA) addition on live body weight at different feeding stages (stage 1, 1–62 day; stage 2, 63–120 day).

As shown in Figure 2, the live body weight of feedlot lambs in both OH and OHWS groups was linearly increased during 120-day feeding. The growth coefficient ranked: CGAA > UGAA > Control in OH group, but the coefficient in OHWS group ranked: UGAA > CGAA > Control, suggesting that both UGAA and CGAA promoted daily gain of the lambs. In addition, regardless the form of GAA was added at day 120 (Supplementary Table 1), the lambs in OH group in comparison with OHWS group presented greater BW (*P* = 0.031), and dietary CGAA in OH group and dietary UGAA addition in OHWS group presented greater BW compared with those of the control group (*P* < 0.05).

The effects of forage type and GAA addition are presented in Table 3. Interaction between forage type and GAA addition was found for the DMI in stage 1, UGAA or CGAA addition in OH group increased DMI than the control (*P* < 0.05), while there was no difference in OHWS group (*P* > 0.05). Regardless the form of GAA was added, the lambs in OH group in comparison with OHWS group presented greater DMI (*P* = 0.003) and greater ADG (*P* = 0.043), but no difference occurred for F: G between the two forage groups. Compared with the control, both UGAA and CGAA did not significantly increase ADG except for somewhat numerical increments. However, both UGAA and CGAA increased DMI (*P* = 0.004). Likewise, no improvement of F: G was found in both UGAA and CGAA additions.

**Table 3:** Effects of forage type and guanidine acetic acid (GAA) addition on growth performance of feedlotting lambs at different feeding stages (stage 1, 1–62 day; stage 2, 63–120 day).

**Items**	**Forage**	**GAA addition**			**S.E.M**	***P*** **-value**		
		**Control**	**UGAA**	**CGAA**		**Forage**	**GAA**	**Interaction**
Feeding stage 1							
DMI (g/day)	OH	915^b^	1051^a^	1036^a^	23.5	0.003	0.004	0.019
	OHWS	933	968	918				
ADG (g/day)	OH	275	287	307	9.6	0.043	0.217	0.233
	OHWS	269	279	270				
F: G	OH	3.36	3.65	3.41	0.115	0.923	0.293	0.546
	OHWS	3.50	3.53	3.41				
Feeding stage 2							
DMI (g/day)	OH	1,430	1,436	1,462	27.3	0.099	0.414	0.949
	OHWS	1,382	1,406	1,424				
ADG (g/day)	OH	300	317	330	13.3	0.206	0.077	0.717
	OHWS	276	316	308				
F: G	OH	4.87	4.66	4.53	0.228	0.612	0.195	0.702
	OHWS	5.10	4.52	4.74				
Whole experiment period							
DMI (g/day)	OH	1,164^b^	1,237^ab^	1,241^a^	22.2	0.010	0.058	0.358
	OHWS	1,150^b^	1,180^a^	1,163^b^				
ADG (g/day)	OH	287^b^	302^ab^	318^a^	8.0	0.022	0.014	0.358
	OHWS	273^b^	297^a^	289^ab^				
F: G	OH	4.08	4.10	3.93	0.119	0.500	0.359	0.537
	OHWS	4.26	4.00	4.05				

OH, the rations with the forage type of oaten hay; OHWS, the rations with the forage type of oaten hay plus wheat silage; UGAA, the rations added with 1 g/kg of uncoated GAA product; the rations added with 1 g/kg of coated GAA product.

ADG, average daily gain; DMI, dry matter intake; F: G, feed conversion efficiency expressed as the ratio of feed intake to live weight gain; S.E.M, standard error of least square means.

a, b In the same row with different superscript letters indicates the significantly different values of P < 0.05.

At the stage 2 unlike the stage 1, neither forage type nor GAA addition significantly alter DMI, and dietary addition of UGAA and CGAA tended to increase ADG (*P* = 0.077). Same as the stage 1, no improvement of F: G was found by both GAA addition and different forage type (Table 3).

For the whole experimental period, the lambs in OHWS group in comparison with OH group presented not only lower DMI (*P* = 0.010) but also lower ADG (*P* = 0.022) though F: G did not differ between the two forage groups. GAA addition (UGAA and CGAA) increased ADG (*P* = 0.014). In addition, dietary CGAA in OH group and dietary UGAA addition in OHWS group presented greater DMI and ADG compared with those of the control (*P* < 0.05). The overall F: G did not alter in response to both forage type and GAA addition (Table 3).

### Effect of forage type and GAA addition on total tract nutrient digestibility

No forage type and GAA addition interaction was observed for nutrient digestibility (Table 4). At the stage 1, the lambs in OH group in comparison with OHWS group presented greater NDF (*P* = 0.013) and ADF (*P* < 0.001) digestibility. At the same time, both UGAA and CGAA increased DMD (*P* = 0.001), OMD (*P* = 0.018), NDFD (*P* = 0.021), and ADFD (*P* < 0.001) digestibility.

**Table 4:** Effects of forage type and guanidine acetic acid (GAA) addition on nutrient digestibility in feedlotting lambs at different feeding stages (stage 1, 58–60 day; stage 2, 118–120 day).

**Items**	**Forage**	**GAA addition**			**S.E.M**	***P*** **-value**		
		**Control**	**UGAA**	**CGAA**		**Forage**	**GAA**	**Interaction**
Stage 1 (58–60)							
DMD (%)	OH	74.01^b^	74.88^b^	77.03^a^	0.543	0.766	0.001	0.443
	OHWS	74.52	74.86	76.14				
OMD (%)	OH	76.84^b^	78.43^ab^	79.15^a^	0.569	0.837	0.018	0.541
	OHWS	77.67	78.06	78.99				
CPD (%)	OH	77.01	77.36	77.30	0.554	0.170	0.824	0.953
	OHWS	76.41	76.53	76.80				
GED (%)	OH	76.07	76.04	76.18	0.453	0.431	0.803	0.946
	OHWS	76.31	76.22	76.65				
EED (%)	OH	83.18	83.35	83.51	0.861	0.645	0.980	0.983
	OHWS	83.01	83.03	83.02				
NDFD (%)	OH	45.21^b^	47.78^a^	48.48^a^	0.600	0.013	0.021	0.051
	OHWS	45.69	45.69	46.05				
ADFD (%)	OH	35.98^b^	40.52^a^	40.71^a^	0.498	<0.001	<0.001	0.113
	OHWS	36.61	36.88	36.99				
Stage 2 (118–120)							
DMD (%)	OH	78.17	78.56	80.39	0.761	0.111	0.047	0.133
	OHWS	78.60^b^	81.44^a^	80.21^ab^				
OMD (%)	OH	81.25^b^	81.57^b^	84.07^a^	0.805	0.200	0.043	0.054
	OHWS	81.72^b^	84.73^a^	83.06^a^				
CPD (%)	OH	78.30	79.02	80.68	0.875	0.701	0.160	0.092
	OHWS	78.66	81.31	78.87				
GED (%)	OH	82.89	82.40	83.03	0.701	0.693	0.964	0.606
	OHWS	82.67	83.45	82.89				
EED (%)	OH	85.41	85.51	86.26	1.209	0.692	0.897	0.955
	OHWS	86.09	86.05	86.25				
NDFD (%)	OH	47.62^b^	51.07^a^	51.45^a^	0.816	0.212	0.025	0.126
	OHWS	48.79	49.29	49.48				
ADFD (%)	OH	40.31^b^	44.29^a^	44.35^a^	0.800	0.966	0.021	0.051
	OHWS	42.81	43.12	43.10				

OH, the rations with the forage type of oaten hay; OHWS, the rations with the forage type of oaten hay plus wheat silage; UGAA, the rations added with 1 g/kg of uncoated GAA product; the rations added with 1 g/kg of coated GAA product.

ADFD, acid detergent fiber digestibility; CPD, crude protein digestibility; DMD, dry matter digestibility; EED, ether extract digestibility; GED, gross energy digestibility; NDFD, neutral detergent fiber digestibility; OMD, organic matter digestibility; S.E.M, standard error of least square means.

a, b In the same row with different superscript letters indicates the significantly different values of P < 0.05.

No difference was observed for nutrient apparent digestibility as an effect of forage type at the stage 2. Similar to stage 1, GAA, whether coated or not, improved DM (*P* = 0.047), OM (*P* = 0.043), NDF (*P* = 0.025), and ADF (*P* = 0.021) digestibility.

### Effect of forage type and GAA addition on blood metabolites

As shown in Table 5, no improvement of the concentration of TP, ALB, urea N, TG, glucose, and IGF-1 was found in two forage-type diets at stage 1. Both forms of GAA resulted in greater TP (*P* < 0.001) and IGF-1 (*P* = 0.004) level, but less urea N (*P* < 0.001), TG (*P* < 0.001), and glucose (*P* < 0.001) concentration in lambs.

**Table 5:** Effects of forage type and guanidine acetic acid (GAA) addition on blood metabolites in feedlotting lambs at different feeding stages (stage 1, 60 day; stage 2, 120 day).

**Items**	**Forage**	**GAA addition**			**S.E.M**	***P*** **-value**		
		**Control**	**UGAA**	**CGAA**		**Forage**	**GAA**	**Interaction**
Stage 1 (60 d)								
TP (g/L)	OH	63.22^b^	71.17^a^	69.26^a^	0.907	0.114	<0.001	0.136
	OHWS	63.57^b^	67.90^a^	68.58^a^				
ALB (g/L)	OH	38.07	38.04	38.12	0.846	0.974	0.316	0.230
	OHWS	38.14	38.49	39.07				
urea N (mmol/L)	OH	13.09^a^	11.67^b^	11.82^b^	0.397	0.371	<0.001	0.749
	OHWS	13.08^a^	11.05^b^	11.57^b^				
TG (mmol/L)	OH	0.44^a^	0.40^b^	0.40^b^	0.009	0.672	<0.001	0.938
	OHWS	0.44^a^	0.41^b^	0.41^b^				
glucose (mmol/L)	OH	4.90^a^	4.50^b^	4.30^b^	0.282	0.745	<0.001	0.453
	OHWS	4.92^a^	4.36^b^	4.36^b^				
IGF-1 (ng/ml)	OH	42.34^b^	44.13^a^	44.38^a^	0.498	0.915	0.004	0.778
	OHWS	42.70	44.01	43.99				
Stage 2 (120 d)								
TP (g/L)	OH	65.03^b^	67.68^a^	67.18^a^	0.783	0.697	0.002	0.585
	OHWS	65.02^b^	67.27^a^	68.35^a^				
ALB (g/L)	OH	39.16	41.10	40.90	1.096	0.178	0.373	0.826
	OHWS	38.57	39.14	39.75				
urea N (mmol/L)	OH	11.43^a^	9.84^b^	9.65^b^	0.453	0.267	0.005	0.573
	OHWS	10.51	9.90	9.25				
TG (mmol/L)	OH	0.50^a^	0.42^b^	0.43^b^	0.017	0.052	0.004	0.176
	OHWS	0.44^a^	0.43^ab^	0.40^b^				
Glucose (mmol/L)	OH	4.61^a^	4.06^b^	4.38^b^	0.227	0.850	0.018	0.450
	OHWS	4.83^a^	4.10^b^	4.03^b^				
IGF-1 (ng/ml)	OH	40.18^b^	44.20^a^	44.18^a^	0.791	0.624	<0.001	0.993
	OHWS	40.60^b^	44.43^a^	44.49^a^				

OH, the rations with the forage type of oaten hay; OHWS, the rations with the forage type of oaten hay plus wheat silage; UGAA, the rations added with 1 g/kg of uncoated GAA product; the rations added with 1 g/kg of coated GAA product.

ALB, albumin; IGF-1, insulin-like growth factor 1; TG, triglycerides; TP, total protein; S.E.M, standard error of least square means.

a, b In the same row with different superscript letters indicates the significantly different values of P < 0.05.

Similar to stage 1, higher serum TP (*P* = 0.002) and IGF-1 (*P* < 0.001), lower urea N (*P* = 0.005), TG (*P* = 0.004), and glucose (*P* = 0.018) levels in lambs on diets are supplemented with GAA (UGAA or CGAA) in stage 2. No significant difference was found for other indicators with GAA addition (*P* > 0.05).

The interaction of forage × GAA was not significant for GAA, creatine, and creatine-related metabolites (Table 6). At stage 1, serum GAA (*P* = 0.001) and CK (*P* < 0.001) levels increased, but GAMT (*P* < 0.001) and AGAT (*P* < 0.001) decreased with UGAA and CGAA supplementations.

**Table 6:** Effects of forage type and guanidine acetic acid (GAA) addition on GAA, creatine, and creatine-related metabolites in feedlotting lambs at different feeding stages (stage 1, 60 day; stage 2, 120 day).

**Items**	**Forage**	**GAA addition**			**S.E.M**	***P*** **-value**		
		**Control**	**UGAA**	**CGAA**		**Forage**	**GAA**	**Interaction**
Stage 1 (60 d)								
CK (U/L)	OH	104.42^b^	121.57^a^	122.48^a^	2.093	0.791	<0.001	0.768
	OHWS	106.11^b^	122.52^a^	121.21^a^				
Creatinine (μmol/L)	OH	64.35	65.19	64.82	1.005	0.232	0.925	0.909
	OHWS	63.78	63.73	63.84				
GAMT (U/L)	OH	17.02^a^	15.76^b^	15.69^b^	0.250	0.668	<0.001	0.942
	OHWS	17.16^a^	15.75^b^	15.84^b^				
AGAT (U/L)	OH	34.33^a^	31.64^b^	31.78^b^	0.451	0.494	<0.001	0.913
	OHWS	34.73^a^	31.98^b^	31.82^b^				
ATP (μmol/L)	OH	1,132.61	1,134.12	1,146.83	20.953	0.656	0.781	0.994
	OHWS	1,143.01	1,140.13	1,153.88				
GAA (μg/mL)	OH	26.32^b^	34.39^a^	33.97^a^	1.458	0.763	0.001	0.830
	OHWS	28.02^b^	34.30^a^	33.62^a^				
Creatine (μg/mL)	OH	22.54	23.86	23.20	0.747	0.097	0.572	0.898
	OHWS	24.14	24.67	24.45				
Stage 2 (120 d)								
CK (U/L)	OH	70.98	75.92	75.23	1.372	0.030	<0.001	0.563
	OHWS	71.85^b^	78.75^a^	79.06^a^				
Creatinine (μmol/L)	OH	55.23^b^	66.14^a^	67.18^a^	1.422	0.645	<0.001	0.688
	OHWS	54.63^b^	66.87^a^	65.43^a^				
GAMT (U/L)	OH	18.36^a^	17.49^b^	17.57^b^	0.308	0.244	0.002	0.196
	OHWS	18.56^a^	16.53^b^	17.40^b^				
AGAT (U/L)	OH	37.99^a^	35.03^b^	35.00^b^	0.690	0.062	<0.001	0.323
	OHWS	37.12^a^	32.93^b^	34.83^b^				
ATP (μmol/L)	OH	1,157.85^b^	1,253.87^a^	1,247.98^a^	26.628	0.879	0.003	0.905
	OHWS	1,148.50^b^	1,268.30^a^	1,253.01^a^				
GAA (μg/mL)	OH	28.54^b^	36.03^a^	36.45^a^	0.730	0.09	<0.001	0.905
	OHWS	29.94^b^	37.21^a^	37.17^a^				
Creatine (μg/mL)	OH	27.06	27.32	27.46	0.385	0.235	0.276	0.795
	OHWS	27.15	27.82	28.07				

OH, the rations with the forage type of oaten hay; OHWS, the rations with the forage type of oaten hay plus wheat silage; UGAA, the rations added with 1 g/kg of uncoated GAA product; the rations added with 1 g/kg of coated GAA product.

AGAT, L-Arginine glycine amidine transferase catalyzes; ATP, adenosine Triphosphate; CK, creatine kinase; GAMT, guanidinoacetate N-methyltransferase; S.E.M, standard error of least square means.

a, b In the same row with different superscript letters indicates the significantly different values of P < 0.05.

At stage 2, the lambs in OH group in comparison with OHWS group presented less serum CK (*P* = 0.030) concentration. In addition, compared with the control, GAA (UGAA or CGAA) increased CK (*P* < 0.001), creatinine (*P* < 0.001), ATP (*P* = 0.003), GAA (*P* < 0.001) levels but decreased GAMT (*P* = 0.002) and AGAT (*P* < 0.001) levels.

### Effect of forage type and GAA addition on blood antioxidant levels

Forage type and GAA addition interacted (*P* < 0.001) to affect SOD, CAT, GSH-*PX*, T-AOC, GSH, and MDA concentrations (Table 7). At stage 1, for lambs in OH group, compared with UGAA and the control, CGAA addition presented higher SOD, CAT, GSH-*PX*, and T-AOC contents (*P* < 0.05), serum concentration of MDA decreased (*P* < 0.05) with UGAA or CGAA supplementation. For lambs in OHWS group, the serum concentrations of SOD and CAT increased (*P* < 0.05) with UGAA or CGAA supplementation. In addition, the concentrations of SOD and CAT were higher (*P* < 0.05) in UGAA than those in CGAA. Serum concentration of MDA decreased (*P* < 0.05) with UGAA or CGAA supplementation, and the concentration of MDA was lower (*P* < 0.05) in UGAA than those in CGAA. At stage 2, lambs on diets supplemented with CGAA had increased (*P* < 0.05) serum concentrations of SOD, CAT, GSH-*PX*, and T-AOC, and decreased MDA (*P* < 0.05) compared with UGAA and the control in OH group. Lambs on diets supplemented with UGAA or CGAA had increased (*P* < 0.05) serum concentrations of SOD, CAT, GSH-*PX*, T-AOC, GSH, and decreased (*P* < 0.05) MDA concentration compared with the control in OHWS group. Concentrations of SOD, CAT, GSH-*PX*, T-AOC, GSH were higher (*P* < 0.05), and MDA was lower (*P* < 0.05) for lambs in UGAA than those in CGAA in OHWS group.

**Table 7:** Effects of forage type and guanidine acetic acid (GAA) addition on serum antioxidant index.

**Items**	**Forage**	**GAA addition**				***P*** **-value**		
		**Control**	**UGAA**	**CGAA**	**S.E.M**	**Forage**	**GAA**	**Interaction**
Stage 1 (60 d)								
SOD (U/ml)	OH	94.18^b^	120.57^b^	181.26^a^	7.602	0.529	<0.001	<0.001
	OHWS	83.79^c^	200.66^a^	123.49^b^				
CAT (U/ml)	OH	7.49^b^	8.07^b^	9.56^a^	0.176	0.646	<0.001	<0.001
	OHWS	7.25^c^	9.91^a^	8.16^b^				
GSH-*PX* (U/ml)	OH	805.07^b^	839.07^b^	923.25^a^	10.607	0.654	<0.001	<0.001
	OHWS	799.20	933.27	846.73				
T-AOC (U/ml)	OH	10.59^b^	11.34^b^	13.19^a^	0.243	0.666	<0.001	<0.001
	OHWS	10.25	13.66	11.48				
GSH (U/ml)	OH	8.42^b^	8.99^b^	10.54^a^	0.188	0.507	<0.001	<0.001
	OHWS	8.18	10.95	9.13				
MDA (nmol/ml)	OH	5.02^a^	4.43^b^	3.00^b^	0.181	0.574	<0.001	<0.001
	OHWS	5.25^a^	2.57^c^	4.37^b^				
Stage 2 (120 d)								
SOD (U/ml)	OH	103.09^b^	116.50^b^	166.68^a^	5.987	0.054	<0.001	<0.001
	OHWS	92.68^c^	185.83^a^	137.00^b^				
CAT (U/ml)	OH	8.46^b^	8.55^b^	9.50^a^	0.138	0.126	<0.001	<0.001
	OHWS	8.10^c^	9.86^a^	9.09^b^				
GSH-*PX* (U/ml)	OH	929.47^b^	946.30^b^	1,005.26^a^	12.538	0.619	<0.001	<0.001
	OHWS	907.69^c^	1,029.11^a^	959.74^b^				
T-AOC (U/ml)	OH	12.09^b^	12.50^b^	13.52^a^	0.182	0.038	<0.001	<0.001
	OHWS	11.92^c^	14.21^a^	12.95^b^				
GSH (U/ml)	OH	10.15^b^	10.56^b^	11.83^a^	0.178	0.385	<0.001	<0.001
	OHWS	9.73^c^	12.31^a^	10.89^b^				
MDA (nmol/ml)	OH	4.37^a^	4.16^a^	3.20^b^	0.119	0.132	<0.001	<0.001
	OHWS	4.59^a^	2.88^c^	3.81^b^				

OH, the rations with the forage type of oaten hay; OHWS, the rations with the forage type of oaten hay plus wheat silage; UGAA, the rations added with 1 g/kg of uncoated GAA product; the rations added with 1 g/kg of coated GAA product.

CAT, catalase; GSH, glutathione; GSH-PX, glutathione peroxidase; MDA, malondialdehyde; SOD, superoxide dismutase; T-AOC, total antioxidant Capacity; S.E.M, standard error of least square means.

a, b, c In the same row with different superscript letters indicates the significantly different values of P < 0.05.

At stage 1 and stage 2, regardless of GAA form, the lambs presented greater (*P* < 0.001) serum SOD, CAT, GSH-*PX*, T-AOC, and GSH, but lower MDA (*P* < 0.001) concentration. However, the lambs in OH group had less T-AOC level than that in OHWS group in stage 2.

## Discussion

### Effect of forage type and GAA addition on growth performance

In the present study, the DMI and ADG of lambs decreased in the OHWS group in comparison with the OH group at stage 1 and the experimental period. This result confirmed that silage (DM) replaces equal amounts of hay to save TMR but reduced ADG. As a result, F: G did not change much (21). However, Wu et al. (22), reported that replacing 20% of the peanut vine hay with foxtail millet silage did not affect the DMI and ADG, while when replacing 60% increased the DMI of dorper-Hu hybrid male lambs. The possible reason was that the raw material of wheat silage selected for this study was at dough stage, and the NDF and ADF contents were lower than that of oaten hay. Lower fiber could reduce ruminal peristalsis, decrease ruminal passage rate, and reversely lower DMI (23).

In previous studies, uncoated GAA was applied as feed additive, and it was found as an improvement of growth performance and feed efficiency in pigs, broiler chickens, and bulls (24–26). However, there was little information available concerning the forage type and GAA addition on Han lamb performance. In addition, regardless the form of GAA was added, the DMI in the stage 1 and ADG in the whole experimental period of lambs were higher than that in the control group. This was consistent with previously reported about Jinjiang bulls and Angus bulls. Li et al. (26) and Li et al. (27) found that the DMI of Jinjiang bulls and Angus bulls increased with GAA addition. However, the findings of the present study contrasted with the results of a previous study with uncoated GAA at Xinjiang Agricultural University of China (28), in which adding GAA without rumen protection treatment in TMR significantly decreased the DMI of lambs but the ADG and F: G were similar. Similar findings were also reported by Liu et al. (11), who found that GAA or coated folic acid had no effect on the DMI of Angus cattle. The differences may be attributed to differences in the initial BW of the experimental animals. The initial BW of lambs was 27 kg in the previous study, but only 12 kg of initial BW was tested in subsequent study. However, no change in DMI, ADG, and F: G of lambs was observed in stage 2 (30–50 kg). This also showed that the effect of GAA in different animals and even in different diets of the same animal was different. In the present study, it was worth mentioning that the GAA content of CGAA was 0.6 g/g, and when the same dose was added, the growth performance of lambs could reach or exceed the addition of UGAA; thus, the coated treatment might be necessary for ruminants.

### Effect of forage type and GAA addition on total tract nutrient digestibility

Similar nutrient digestibilities were observed for lambs fed two types of forages except for NDF and ADF in stage 1. Lambs fed OH diet had higher digestibility of NDF and ADF than OHWS diet, this corresponds to higher DMI in OH diet. These results were consistent with those from other reports that the DMI of cows was positively related to dietary NDF digestion (29). In addition, the digestibility of NDF and ADF can be used to measure the digestibility of diets in ruminants (30), but other nutrient digestibilities did not significantly increase except for somewhat numerical increments. This may be related to the proportion of oat hay in OHWS diet in this study.

Li et al. (27) and Liu et al. (11) observed increased apparent digestibilities of DM, OM, CP, NDF, and ADF with supplementing GAA in diets of bulls. In agreement with previous research, the present study observed increased apparent digestibilities of DM, OM, NDF, and ADF with supplementing UGAA and CGAA at two stages. The increment in ADG was probably associated with the increase in total-tract nutrient digestibility with UGAA or CGAA supplementation. Also, the addition of GAA could improve intestinal cellular energy metabolism (31) and gut morphology (25). Therefore, the addition of UGAA or CGAA to lamb diets may play a role in improving post-ruminal nutrient digestion.

### Effect of forage type and GAA addition on blood metabolites

The current research results showed that the forage type did not affect serum metabolic indexes. This also revealed that it is feasible to replace a certain proportion (36%) of OH with OHWS.

Dietary UGAA or CGAA addition increased serum TP and IGF-1 in stage 1 and stage 2. The change was in accordance with that in Angus bulls (11). The increase in the concentration of IGF-1 corresponds to the increased growth performance of UGAA or CGAA group. A limited response of serum glucose, urea N, and TG with the addition of GAA or CGAA in stage 1 and stage 2 was consistent with the findings in mice (32), indicating that supplementary UGAA or CGAA affected insulin homeostasis and ameliorates hyperglycemia. The reduction in TG levels also provided indirect evidence that GAA reduces fat deposition (33). However, Speer et al. (13), reported that the plasma urea N level increased with continuous abomasal infusion of 7.5 or 15 g/day GAA in steers. The different supplementation modes and dose of UGAA or CGAA in the two studies could explain the inconsistent results.

The formation of GAA is normally the rate-limiting step of creatine biosynthesis, the AGAT reaction is the control step in the pathway. AGAT can be feedback inhibited by GAA (34). Therefore, decreased serum AGAT activity was observed with UGAA or CGAA addition at stage 1 and stage 2. GAMT is responsible for catalyzing the transfer of the methyl group from S-adenosylmethionine to GAA to form creatine and S-adenosylhomocysteine, S-adenosylhomocysteine has an inhibitory effect on the activity of GAMT (34). The negative response of blood GAMT activity was consistent with the reaction with the GAA decomposition into creatine with UGAA or CGAA addition. In this study, serum CK activity and creatine concentration increased significantly after GAA addition, which also proved that CK activity was highly correlated with creatine concentration (34). Furthermore, dietary GAA (1 g/kg UGAA or CGAA) increased blood GAA concentration in the present study. It was also observed that the GAA content was increased in the blood of Kazakh male lambs when 0.5 or 1 g/kg GAA was supplemented in the diets (28), which was consistent with our study. But the blood creatine concentration was not increased, which was inconsistent with previous studies (28, 35), and it might be attributed to differences in dietary GAA dosage (0.5 g/kg GAA in lambs diet; 0.3 g/kg GAA in pigs diet).

### Effect of forage type and GAA addition on blood antioxidant levels

Although the effect of forage type on the antioxidant capacity of lamb serum was limited, forage type and GAA addition interacted to affect SOD, CAT, GSH-*PX*, T-AOC, GSH, and MDA concentration. Because the only difference between the two forage diets was that part of oaten hay is replaced by equal wheat silage (DM), the possible reason was that the CGAA was partially degraded by wheat silage-related microorganisms in the rumen, resulting in a decrease in the amount of blood absorbed into the intestine after entering the rumen.

At present, the antioxidant effect of GAA is inconsistent, generally speaking, the appropriate concentration of GAA has antioxidant effect, and excessive GAA may generate a hydroxyl radical, strong free radical, and impede antioxidant capacity (36). The antioxidant process is actually the process of removing excess free radicals. A process that involves many antioxidant enzymes which must be precisely coordinated. The antioxidant defense mechanism of the body is a set of antioxidant enzyme systems, including T-AOC, CAT, and GSH-*PX*, which can protect the body from the damage of reactive oxygen species (37). SOD is responsible for the breakdown of superoxide anions into hydrogen peroxide, while CAT and GSH-*PX* can reduce hydrogen peroxide, thereby preventing the production of highly toxic hydroxyl-free radicals. Importantly, GSH-*PX* can also reduce hydrogen peroxides of polyunsaturated fatty acids, thereby counteracting the toxic effects of lipid peroxide. GSH has antioxidant and integrated detoxification effects. T-AOC used to reflect the total capacity of antioxidant systems The final product of lipid peroxide is MDA. In our study, with the addition of UGAA or CGAA, SOD, CAT, GSH-*PX*, T-AOC, and GSH increased, the MDA decreased, and in OHWS diet was more obvious (in stage 1 and stage 2). This is consistent with previous studies on pigs (38). It is worth mentioning that there is a clear interaction between the two forage type diets addition UGAA or CGAA. This also suggested that producers choose different type of GAA or increase the amount of CCAA added for different forage composition.

## Conclusion

Under a high-concentrate feedlotting pattern, the feed results obtained in the present study indicated that daily gain presented a greater increase response to not only UGAA but also CGAA supplementation in young lambs with body weight from 13 to 30 kg, and such positive responses were more pronounced in oaten hay fed lambs in comparison with oaten hay plus wheat silage fed lambs. However, feed efficiency (F: G) was not improved through feed intake tended to increase in response to both UGAA and CGAA additions. In subsequent feedlotting body weight increased from 30 to 50 kg, and dietary addition of whatever form of GAA did not show a beneficial response to growth performance though the GAA addition somewhat increased dietary DM, OM, and fiber (e.g., NDF and ADF) digestion. Both UCGA and CGAA additions increased IGF-1 level, CK activity, as well as antioxidant capacity, but decreased GAMT and AGAT activities in the blood. Taken together, the results obtained in the present study implicated GAA could be applied to improve growth performance in younger (13–30 kg) instead of older (30–50 kg) in feeding practice.

## Data availability statement

The original contributions presented in the study are included in the article/Supplementary material, further inquiries can be directed to the corresponding author.

## Ethics statement

The animal study was reviewed and approved by Beijing Municipal Council on Animal Care (with protocol CAU20171014-1). Written informed consent was obtained from the owners for the participation of their animals in this study.

## Author contributions

W-JL and H-JY designed the experiment. W-JL, Z-YC, Y-WJ, AA, Q-CW, FZ, and H-WC conducted the experiment and collected samples. L-KL, F-LX, and Y-YL helped to detect samples. W-JL wrote the first draft. S-LL and H-JY revised the manuscript. All authors contributed to the article and approved the submitted version.

## Funding

This work was supported by the Feed Evaluation and Feed Table Establishment for Meat Sheep project funded by the Ministry of Agriculture and Rural Affairs of the People's Republic of China (grant no. 31572432).

## Conflict of interest

The authors declare that the research was conducted in the absence of any commercial or financial relationships that could be construed as a potential conflict of interest.

## Publisher's note

All claims expressed in this article are solely those of the authors and do not necessarily represent those of their affiliated organizations, or those of the publisher, the editors and the reviewers. Any product that may be evaluated in this article, or claim that may be made by its manufacturer, is not guaranteed or endorsed by the publisher.
