# Supplementary material for: Guanidine acetic acid exhibited greater growth performance in younger (13–30 kg) than in older (30–50 kg) lambs under high-concentrate feedlotting pattern
Source: Front Vet Sci. 2022 Aug 4;9:954675. doi: 10.3389/fvets.2022.954675 (PMC9386046; doi:10.3389/fvets.2022.954675)
Supplement: Supplementary file 1 [file Table_1.docx]

Supplementary Material

# Supplementary Table

**Supplement Table 1** Effects of forage type and GAA addition on live body weight change in feedlotting lambs throughout whole feed period.

| Items | TYPE | GAA | | | SEM | *P*-value | | |
| --- | --- | --- | --- | --- | --- | --- | --- | --- |
|  |  | Control | UGAA | CGAA |  | TMR | GAA | TYPE×GAA |
| Initial BW (kg) | OH | 13.0 | 13.2 | 13.0 | 0.57 | 0.806 | 0.888 | 0.975 |
|  | OHWS | 13.0 | 13.1 | 12.8 |  |  |  |  |
| Final BW (kg) | | | | | | | | |
| d18 | OH | 17.0 | 18.1 | 18.0 | 0.77 | 0.514 | 0.676 | 0.697 |
|  | OHWS | 17.3 | 17.6 | 16.9 |  |  |  |  |
| d33 | OH | 21.4 | 23.4 | 22.8 | 0.93 | 0.317 | 0.309 | 0.844 |
|  | OHWS | 21.2 | 22.2 | 21.7 |  |  |  |  |
| d47 | OH | 25.4 | 27.0 | 27.0 | 0.96 | 0.199 | 0.362 | 0.913 |
|  | OHWS | 24.8 | 25.8 | 25.6 |  |  |  |  |
| d62 | OH | 30.1 | 31.5 | 32.0 | 0.94 | 0.133 | 0.456 | 0.561 |
|  | OHWS | 29.6 | 30.6 | 29.6 |  |  |  |  |
| d90 | OH | 38.0 | 39.9 | 40.2 | 0.99 | 0.053 | 0.171 | 0.584 |
|  | OHWS | 36.9 | 38.9 | 37.3 |  |  |  |  |
| d120 | OH | 47.5^b^ | 49.8^ab^ | 51.1^a^ | 1.03 | 0.031 | 0.030 | 0.494 |
|  | OHWS | 45.7^b^ | 48.9^a^ | 47.4^ab^ |  |  |  |  |
